# Direct Glass-to-Metal Welding by Femtosecond Laser Pulse Bursts: II, Enhancing the Weld Between Glass and Polished Metal Surfaces

**DOI:** 10.3390/nano15161215

**Published:** 2025-08-08

**Authors:** Qingfeng Li, Fei Luo, Gabor Matthäus, David Sohr, Stefan Nolte

**Affiliations:** 1 Institute of Applied Physics, Abbe Center of Photonics, Friedrich Schiller University Jena, Albert-Einstein-Str. 15, 07745 Jena, Germany; qli@thorlabs.com (Q.L.); fei.1.luo@kcl.ac.uk (F.L.); gabor.matthaeus@zeiss.com (G.M.); stefan.nolte@uni-jena.de (S.N.); 2 Thorlabs GmbH, Münchner Weg 1, 85232 Bergkirchen, Germany; 3 Department of Engineering, King’s College London, Strand Campus, Westminster WC2R 2LS, UK; 4 ZEISS Photonics & Optics, ZEISS Microoptics, Carl Zeiss Jena GmbH, Carl-Zeiss-Promenade 10, 07745 Jena, Germany; 5 Fraunhofer Institute for Applied Optics and Precision Engineering IOF, Albert-Einstein-Str. 7, 07745 Jena, Germany

**Keywords:** ultrashort laser pulses, micromachining, bonding, glass-to-metal welding

## Abstract

We present a comprehensive study on the femtosecond laser direct welding of glass and metal, focusing on optimizing processing parameters and understanding the influence of material properties and beam shaping on welding quality. Using microscopy, we identified optimal pulse energy, focal position, and line-spacing for achieving high-quality welds. We further investigated the effects of laser beam shaping and material property differences in various glass-to-metal pairs, including borosilicate, fused silica, and Zerodur glasses welded with mirror-polished metals such as Cu, Mo, Al, Ti, and AISI316 steel. Our results show that Ti and AISI316 steel exhibit the lowest adhesion to borosilicate and fused silica glasses, while Zerodur glass achieves good adhesion with all tested metals. To understand the weldability differences among material pairs, we employed a time-dependent finite-element method to analyze the laser heating-induced thermal stress. Our findings indicate that the welding quality is significantly influenced by the choice of materials and beam shaping, with the vortex beam showing potential for improved welding outcomes. This study provides valuable insights for optimizing glass-to-metal welding processes for various industrial applications.

## 1. Introduction

This paper forms part II of our mini-series on glass-to-metal welding within this special edition. When studying the welding onto unpolished metal surfaces in part I [1], we found that the impact of the gap size between glass and metal leads to such a great range in the resulting weld strength values that the influence of other parameters cannot be quantified with statistical significance. In this second part, we remove the influence of the gap by using mirror-polished metal samples and fixing the assembly parts with a specifically designed clamping system. For each welding experiment, the samples are pressed together so that the first-order Newton ring covers the full to-weld area. With this approach, the glass and metal samples have already achieved close contact before welding, i.e., the distance is much smaller than the critical gap size of 1.5 µm that we identified in part I as the upper limit for a successful weld of borosilicate glass. This way, the influence of other process parameters can be examined quantitatively and with statistically meaningful results. As in part I, we examine the influence of pulse energy and longitudinal focus position. Additionally, we consider the effect of the spacing between welding lines.

For an explanation of the fundamental bonding mechanisms in femtosecond-laser direct welding, please see Section 1 in part I [1].

Since Tamaki et al. originally demonstrated direct welding by femtosecond laser pulses [2], Gaussian beams have been widely used in ultrashort pulsed laser welding of brittle materials (e.g., glass, ceramic [3]) and dissimilar materials (e.g., glass–metal [4], glass–silicon [5], glass–SiC [6], and silicon–metal [7]). Recently, to optimize welding qualities for various application scenarios, researchers have explored the possibility of welding with temporally and spatially shaped laser beams. Zhang et al. used the Bessel beam to relax the strict requirement on precisely controlling the focal position [8]. Similarly, other studies have highlighted the benefits of employing an elongated focal shape for glass-to-glass [9] and glass-to-metal welding [10]. Hecker et al. used the average power modulated and spatially shaped laser beam to prevent the irregular formation of weld seams [11]. Here, we compare Gaussian beam welding with optical-vortex (OV) laser welding, motivated by previous findings that identified OV as a favorable beam shape for homogeneous heating and the ability to create unique material structures [12,13].

In the latter part of this paper, we concentrate on investigating how different material pairs influence the results of welding. In recent years, many researchers have demonstrated glass-to-metal welding [4,10,14,15,16,17,18], but the influence of specific physical properties of material pairs on the welding quality is not yet well understood. To further improve the understanding of the weldability of different material pairs, we carried out welding experiments for three types of glasses and five types of metals. In addition to the experimental results, we use a time-dependent finite-element method analysis to evaluate the thermal stress induced during the welding process. Based on the combination of experimental and numerical results, we further constrain the critical damage mechanisms of the welded samples.

## 2. Experimental Setup

For the experiments, we use the same laser processing and characterization systems as in part I [1]. We also use the same Trumpf TruMicro 2030 Femto Edition laser with a wavelength of 1030 nm and a pulse duration of 400 fs. In Section 3.1, Section 3.2 and Section 3.3, we use a burst mode operation with eight pulses at a burst repetition rate of 125 kHz as in part I. Due to technical issues, we had to change to a four-pulse burst mode with a burst repetition rate of 330 kHz for the experiments presented in Section 3.4 and Section 3.5. During our experiments in this part, we use both a 4× (Olympus, PLN, NA = 0.10) and a 10× objective lens (Mitutoyo, M Plan Apo, NA = 0.26) to focus the laser beam. When studying the influence of different beam shapes in Section 3.4, we use a spiral phase plate (SPP) to generate a so-called vortex beam. Our SPP generates an azimuthal phase distribution $\phi \left(\theta \right)=e^{-i\theta }$, which corresponds to a topological charge of −1 and is discretized in 12 steps.

**Figure 1:**
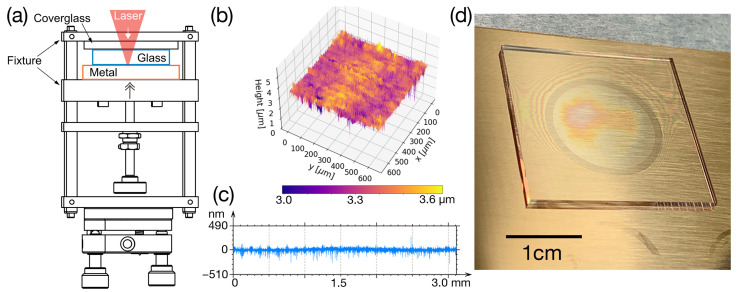
(**a**) The sample clamping system designed for glass-to-metal welding. (**b**) The surface roughness of a mirror-polished metal measured using a confocal laser scanning microscope (CLSM). (**c**) The roughness profile of the a scanned trace. (**d**) A 25 mm × 25 mm × 1 mm glass welded to mirror-polished copper showing the interference fringes (Newton rings) that are indicative of the gap size between glass and metal.

Unlike the free-stacking configuration described in Part I, a sample clamping system, as shown in Figure 1a, is employed here to secure the glass onto the metal sample and ensures close contact between the two materials. Each metal sample has been mirror-polished. A typical surface roughness of those polished metal surfaces is presented in Figure 1b,c. The arithmetic average of the measured roughness profile Ra is 19.3 nm, and the maximum peak-to-valley height of the profile Rz is 343 nm. As an example of the resulting gaps after welding, Figure 1d shows a borosilicate glass plate welded onto mirror-polished copper. Inside the weld seam (visible as a dark ring), we only see first-order interference colors, i.e., the distance between glass and metal in this region is less than half a micrometer.

In addition to borosilicate glass and copper, we also examine silica, Zerodur, and Cu, Mo, Al, Ti, and AISI316 steel as potential welding partners. These are all oxide glasses and oxidizable metals, respectively. Therefore, they can potentially form strong welds through mixed ionic-covalent bonding, as explained in the paragraph “Conditions for Successful Welding” in Section 1 in part I [1]. In Table A1, we list the relevant physical properties of all tested materials.

## 3. Results

This study encompasses an extensive series of welding experiments. To minimize the time required for these investigations, a detailed parametric study is conducted on the effects of pulse energy, focal position, and line spacing, as presented in Section 3.1, Section 3.2 and Section 3.3. The optimized conditions identified in these sections are then applied in Section 3.4 and Section 3.5 to examine the influence of beam shaping and variations in material pairings, respectively. Due to the large number of different input parameter combinations tested in this study, repeated measurements of the breaking force have only been performed for selected data points. In these cases the error bars representing the standard deviation are shown in the graph, e.g., see Figure 3a. As the rest of the data points each represents a single measurement, our results should be considered as a lower bound for the maximum welding strength that can be reached. A more complete representation of the breaking strength would be given by a probability distribution such as the Weibull distribution [14,19].

### 3.1. The Effect of the Pulse Energy

In part I, we determined the processing windows in terms of the input pulse energy for BOROFLOAT^®^ 33 glass (B33) to copper welding when an air gap is present. In this paper, given the assembling condition has changed, we expect to find a new processing window. Therefore, starting from the same burst and focus conditions (125 kHz, eight pulses/burst, NA = 0.26), we first investigate the influence of the input pulse energy on B33-to-Cu welding. In order to gain a better insight into the generalizability of the results, we also performed the same parameter tests for the welding of UV-Grade fused silica (${\mathrm{SiO}}_{2}$) to Cu.

**Figure 2:**
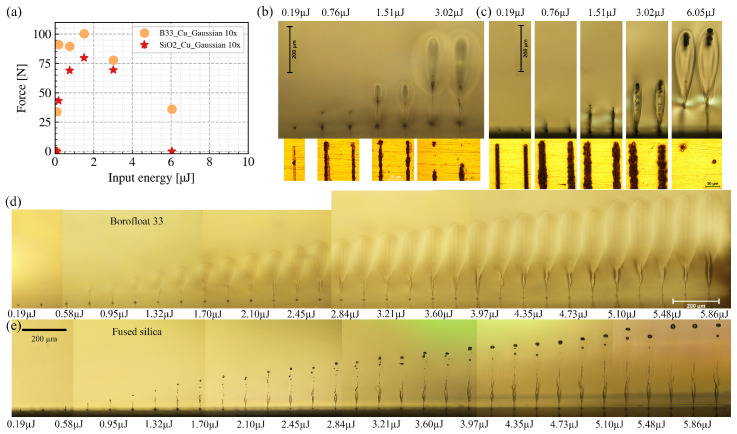
(**a**) Relationship between the input pulse energy and the welds’ breaking force of B33–Cu and ${\mathrm{SiO}}_{2}$–Cu combinations. (**b**,**c**) Side views (upper row) and top views (lower row) of the welding seams under different input pulse energies. (**d**,**e**) Side views of the single site modifications induced near the glass–copper interface under different input pulse energies. Each site was irradiated by 10,000 pulses.

Figure 2a shows how the breaking force of the weld is influenced by the input pulse energies $E_{in}$. For all cases presented here, the focus is positioned at the glass-to-metal interface, the processing speed $v_{p}$ is 1 mm/s, the welding area *A* is 4.5 mm × 2 mm, and it was hatched using a bi-directional scanning strategy with a line interval of 100 µm.

For B33-to-Cu welding, as the input pulse energy increases from low to an intermediate value (e.g., 1.51 µJ), the breaking force increases correspondingly. However, when the input pulse energy further increases, the breaking force starts to decrease. Similar trends apply to the ${\mathrm{SiO}}_{2}$-to-Cu welding; when the input pulse energy increases to 6.05 µJ, the breaking force decreases to 0 N (i.e., ${\mathrm{SiO}}_{2}$ is not welded to Cu in this case).

To understand the mechanism of such relations, we carried out the welding experiments under the same processing conditions with side-polished glasses. After the welding, we selected four typical input energies and performed ex situ side and top observations of the seams. The upper half of Figure 2b shows the modifications in B33 under different input energies, and the lower half shows the modifications at the B33–Cu interface. As the input energy increases, the volume of the in-glass modification increases. However, the modified line width at the B33–Cu interface follows another trend, i.e., from 1.51 µJ to 3.02 µJ; as the energy increases, the modified line width decreases. Specifically, when the input energy increases to 3.02 µJ, the previously continuous line becomes discontinuous, and consequently, the breaking force of the welds decreases. Figure 2c shows the same study on ${\mathrm{SiO}}_{2}$-to-Cu welding. The previously described empirical relation also applies here. Moreover, when the input energy increases to 6.05 µJ, modifications at the interface are almost fully suppressed, and most of the laser energy is absorbed in the bulk of ${\mathrm{SiO}}_{2}$.

In Figure 2d,e, we further investigate the laser-induced modifications near the B33/${\mathrm{SiO}}_{2}$-to-Cu interface for incrementally increasing input pulse energies (step size 0.19 µJ). To better visualize in side view, we chose to have single-site modifications, and to mimic the welding condition, the number of pulses per site was set to 10,000 (125 kHz, eight pulses/burst). The focus was positioned at the interface for all measurements. When the pulse energy is low (e.g., 0.19 µJ), the absorption occurs only within the Rayleigh range of the laser beam, and the modification is confined near (<10 µm) the interface. As the pulse energy increases (e.g., 2.45 µJ), the modification grows far beyond the Rayleigh range of the laser beam; in other words, absorption occurs outside of the focal region. When the input pulse energy is high (e.g., 5.86 µJ), the modification grows even further beyond the focal region. In this case, most of the laser energy is absorbed in the bulk of the glass, and there are no more modifications on the copper surface. To summarize, we have observed a shift of the absorption region towards the incoming laser direction as the input pulse energy increases. This phenomenon has been studied by many groups [7,20,21,22,23,24] and can be explained as follows. For the first laser pulse, the energy of the pulse is absorbed by electrons in the valence band to generate free electrons by photonionization and cascade ionization. However, the photonionization-induced plasma defocusing and the optical Kerr effect shift the maximum intensity position from the linear focal position. For B33 and ${\mathrm{SiO}}_{2}$, in our tested energy range, this shift is small (<5 µm). For later pulses, however, heat accumulation and a temperature rise in the material can lead to increased ionization. When the temperature of the ‘hot area’ reaches 3000 K or more, the pulse energy is almost linearly absorbed, and the glass becomes even opaque. For our experimental conditions, heat accumulation results in peak temperatures of up to several thousand degrees, as calculated by the thermal model that we use for the calculation of thermal stress in Section 3.6. These findings agree with previous experimental observations [25]. At those temperatures, thermal ionization becomes crucial for producing high-density free electrons, which serve as the initial free electrons for the plasma formation induced by subsequent pulses. The teardrop-shaped modifications beyond the Rayleigh range of the laser beam can be explained in terms of the thermal ionization front that moves towards the laser source as the temperatures increase. It is worth noting that the geometrical shape of the modifications is different in B33 and ${\mathrm{SiO}}_{2}$. With the same input pulse energy, the width of the modifications in the B33 is about twice that of ${\mathrm{SiO}}_{2}$. This can be briefly attributed to the fact that the band-gap energy of B33 (4 eV) is lower than that of ${\mathrm{SiO}}_{2}$ (9 eV) [26].

### 3.2. The Effect of the Focal Position

In the last section, we observed that, as the input pulse energy increases, the center of the laser-induced modification shifts upstream from the geometrical focus position towards the laser source. In this section, we investigate the influence of the longitudinal focus-to-interface displacement Δz on the welding quality.

**Figure 3:**
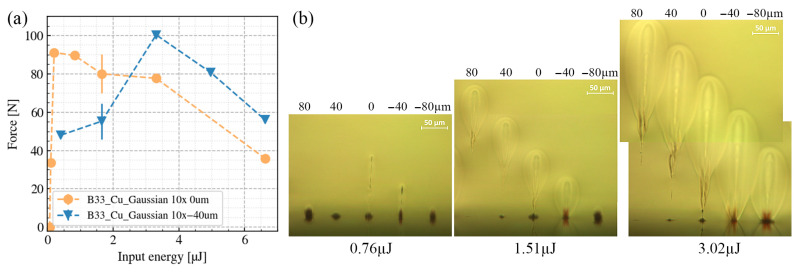
(**a**) Relationship between the longitudinal focus position and the welds’ breaking force of B33–Cu when the focus-to-interface displacement is set at 0 µm and −40 µm. (**b**) Side views of the the single-site modifications under three different input pulse energies (0.76, 1.51, and 3.02 µJ). For each energy, the focus-to-interface displacement is set at 80, 40, 0, −40, and −80 µm. Each site was irradiated with 1250 bursts, each consisting of 8 pulses at the specified pulse energy.

Figure 3b shows the side observations of the single-site modifications under different pulse energies and different Δz. Under the high-pulse-energy conditions (e.g., 3.02 µJ), when the focal position is 80 µm above the interface, a tear-drop shaped modification is confined in the bulk of B33. When the focal position coincides with the interface, the tear-drop-shaped modification extends to the interface as well; however, most of the modification remains inside the glass. By further moving the focal position to 80 µm below the interface, the modification at the interface covers a larger width; however, the volume of the tear-drop-shaped modification in B33 decreases.

Under the intermediate-pulse-energy conditions (e.g., 1.51 µJ), when the focal position is 80 µm above the interface, an in-bulk tear-drop-shaped modification can still be induced. The same applies for the 0 µm case; however, the tear-drop-shaped modification disappears as the focal position shifts 80 µm below the interface.

Under the low-pulse-energy conditions (e.g., 0.76 µJ), when the focal position is above the interface, the pulse energy is not sufficient to generate the tear-drop-shaped modification in the bulk of B33. However, given the fact that copper has a lower laser-induced damage threshold than B33 [1], interface ablation occurs even under the +80 µm conditions. Surprisingly, such ablation is prevented in those intermediate- and high-energy conditions. When the focal position shifts to the interface or 40 µm below, laser-induced free electrons at the Cu-B33 interface act as a seed and induce further laser energy absorption in the bulk of B33.

Despite the differences in the laser-induced modification phenomena under the different focal conditions, we are more concerned about the quality of the welding seams. Figure 3a shows how the breaking force of the welding varies against the input pulse energies under different focal conditions. The orange curve indicates the condition when the focal position coincides with the interface, and the blue curve indicates the condition where the focal position is 40 µm below the interface. From the two curves, one can see that the maximum breaking force for the two focal conditions is similar. However, for the de-focused condition, to achieve a similar breaking force, higher input energy is required. As shown in Figure 3a, a higher input pulse energy ultimately degrades the welding quality. This can be understood as the combined action of the mismatch in thermal expansion between glass and metal and the increased thermal load at higher pulse energies. Together they can lead to excessive internal stress in the glass leading to crack formation. This is in contrast to findings for glass-to-glass welding, where an increase in energy can suppress the formation of cracks [27]. For the subsequent studies, we chose to align the focal position with the glass-to-metal interface.

### 3.3. The Effect of the Line Spacing

As one may have noticed, in the previous sections, we have only mentioned the breaking force rather than the breaking strength of the welding. Conventionally, the breaking strength σ of the welding seams is calculated according to this relation: $\sigma =\frac{F}{A}$. The breaking force *F* can be experimentally measured; however the cross-section area *A* has different definitions. In some definitions, this area only refers to the welding seams covered regions, and the blank intervals between the seams are excluded. In other definitions, the whole welding affected regions (include the seams and the blank intervals) are defined as the cross-section area. By using the second definition, the size of the blank interval (or in another term, the welding seams density) has a strong influence on the breaking strength σ.

**Figure 4:**
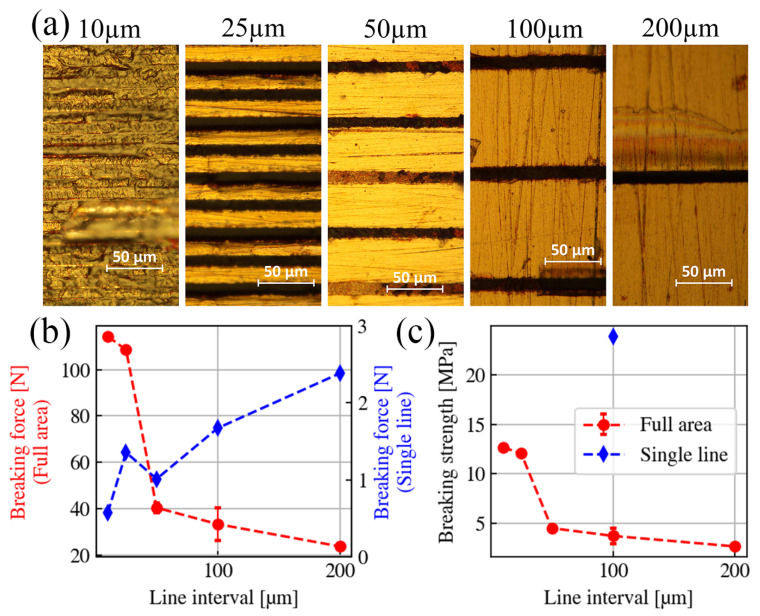
(**a**) Top views of the welding seams under different line spacing conditions. Except for the line spacing, the other processing parameters remain the same: the input pulse energy is 0.38 µJ, the scan speed is 1 mm/s, and the processing area is 4.5 mm × 2.0 mm. (**b**) Relationship between the line spacing and welds’ breaking force. (**c**) Relationship between the line spacing and welds’ breaking strength.

In the aforementioned investigations, the laser welding process is hatched by a bi-directional scanning strategy. Therefore, for certain welding-affected regions, the blank intervals are directly linked to the line spacing Δr. In Figure 4a, under the constant laser processing parameters (125 kHz, eight pulses/burst, NA = 0.26, $E_{in}$ = 0.38 µJ, Δz = 0 µm, and $v_{p}$ = 1 mm/s), we present the top views of the welding seams under different line spacing values (10–200 µm). As shown in the microscopic images, when Δr≥ 100 µm, the width of the processed lines is constantly 13 µm. However, as Δr≤ 25 µm, the processed lines start to interfere with each other. When Δr= 25 µm, the width of the first line is still 13 µm, but the second following line width shrinks to 10 µm, and the third returns to 13 µm again. Under this line spacing condition, a ’breathing’ effect occurs during the hatching. When the line space further decreases to 10 µm, the welding-affected area is fully covered by the seams, and there are no more blank intervals. For all cases, the welding-affected region is fixed at 4.5 mm × 2 mm. Under this condition, the breaking forces for each line spacing condition are plotted in Figure 4b in the red curve. By dividing those measured breaking forces by the number of lines, we obtained the force contributions of a single welding line and plotted them in the blue curve. The red curve shows an intuitive trend which indicates that when the line space gets closer, the breaking force increases. However, the blue curve indicates that the force contributed by a single welding line follows a contradictory trend. As the lines become denser, the force induced by a single line decreases due to the interference.

Finally, we divided the breaking force by the area (4.5 mm × 2 mm) and presented the breaking strength in Figure 4c. According to the second definition of the welding area, the maximum breaking strength (12.7 MPa) is achieved when the line space is set at 10 µm, i.e., the lowest line spacing within our measurement range. However, the welding force per line decreases for smaller line spacing, and thus, the process efficiency is reduced. If we calculate the breaking strength induced by a single welding line under the condition of Δr= 100 µm, the breaking strength induced by a single line is 23.9 MPa. In practice, better hatching strategies should be applied to reach this limit value. However, finding such a strategy is beyond the scope of this paper. In the rest of this paper, we kept the line space at 100 µm and quantified the welding quality by the breaking force.

### 3.4. The Effect of the Beam Shape

**Figure 5:**
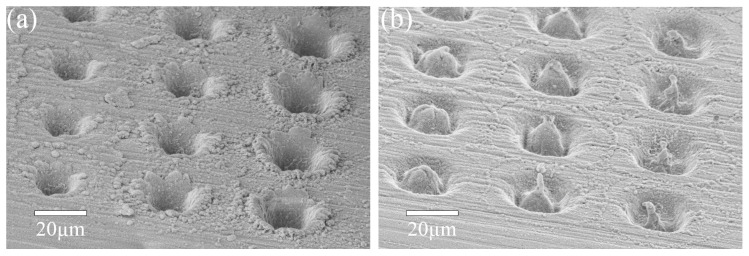
SEM images of ablation sites induced directly on the polished Cu surface without overlying glass by tightly focused (10× MO) (**a**) Gaussian beams and (**b**) vortex beams. Within the same column, the applied pulse energies are identical. From left to right, the three applied pulse energies are 7.56 µJ, 8.82 µJ, and 10.08 µJ. For each ablation site, the applied number of pulses is 20.

Beam shaping is another important control parameter in the pursuit of process optimization. An annular beam, which has an intensity distribution concentrated in a ring with no on-axis intensity, has important implications for thermal and mechanical effects in materials [13,28,29]. In this paper, our annular beam (optical vortex) is generated by a spiral phase plate. From Figure 5, we discovered that, compared to the Gaussian beam, the vortex beam helps achieve inward movements of the molten copper. As the inward displaced molten copper solidifies, the height of the central needles can reach micrometer heights. In this section, we aim to discover if the unique feature of the optical vortex ablation can enhance the welding qualities.

**Figure 6:**
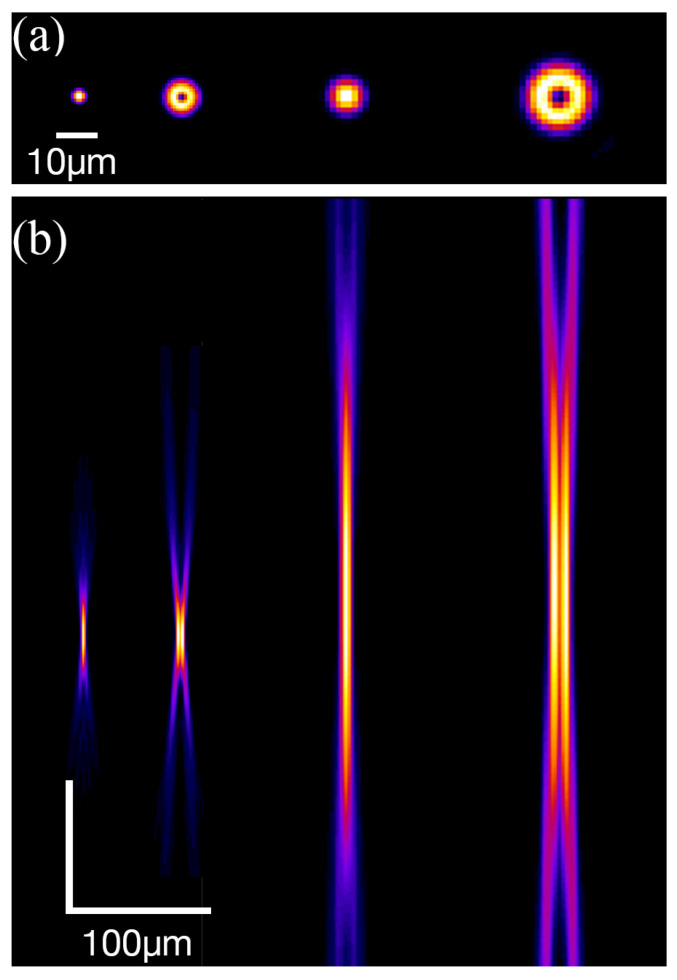
The shape of the beams under different focusing conditions. From left to right, the focal conditions are NA 0.26 Gaussian, NA 0.26 vortex, NA 0.10 Gaussian, and NA 0.10 vortex. (**a**) The normalized intensity distribution in the x–y plane of the intensity-maximized focal plane. (**b**) The normalized intensity distribution in the x–z plane.

As shown in Figure 6, from left to right, in this paper we tested four different beam shapes: tightly focused (10× MO, NA=0.26) Gaussian beam, tightly focused vortex beam, loosely focused (4× MO, NA=0.10) Gaussian beam, and loosely focused vortex beam. Utilizing our benchmarked linear propagation model [30], which accounts for aberrations, the intensity distribution near the focus can be simulated. Figure 6a illustrates the intensity distribution on the x–y plane at the beam waist, and Figure 6b illustrates the intensity distribution along the x–z plane near the focus. For the tightly focused Gaussian beam, the beam radius at the beam waist is 1.9 µm. Under the same focusing condition, the outer ring radius of the vortex beam is 3.5 µm. For the loosely focused Gaussian beam, the beam radius is 4.7 µm. Under the same focusing condition, the outer ring radius of the vortex beam is 8.7 µm.

The main aim of the paper is to investigate how different materials (listed along with their thermal and mechanical properties in the appendix in Table A1) and beam shapes influence welding quality. Such an investigation involves tremendous numbers of parameters; therefore, it is generally time-consuming. To reduce time costs and make such an investigation feasible, based on what is presented in Section 3.1, Section 3.2 and Section 3.3, in the rest of this paper, we decided to perform parameter studies under the following constraints: $E_{in}\leq$ 4.0 µJ, Δz = 0 µm, Δr = 100 µm, $v_{p}$ = 3.3 mm/s, and *A* = 4.5 mm × 2.0 mm. Due to technical reasons we had to change the burst configuration for the following studies. From here on, we use four pulses per burst at a burst repetition rate of 330 kHz; the pulse interval within a burst is still 20 ns.

**Figure 7:**
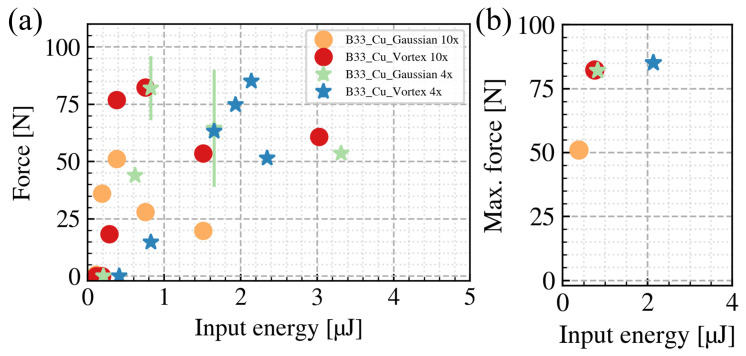
(**a**) Breaking force to input pulse energy relations under different beam shaping conditions. (**b**) The maximum breaking force for each beam shaping condition.

Figure 7a shows the relation between the input pulse energies and the breaking force of the B33-to-Cu welds under different beam shaping conditions. The maximum breaking force for each condition is summarized in Figure 7b. For the tightly focused condition, the maximum breaking force achieved by the vortex beam increased by 61% compared to the Gaussian beam. For the loosely focused condition, there is only a 4% increase. According to our results, it appears that the vortex beam can enhance the welding quality. However, one must also note that, even using the same focusing lens, the effective beam area of a Gaussian beam differs from a vortex beam. Therefore, even though the welding results achieved by the vortex beam show a higher breaking force, we cannot simply attribute this superiority to the unique feature of the vortex beam. Coincidentally, as shown in Figure 6a, the effective beam areas are similar for the tightly focused vortex beam and the loosely focused Gaussian beam. Comparing the maximum breaking force of the two conditions, the values are also very similar: 82.3 N for the tightly focused vortex and 82.0 N for the loosely focused Gaussian. These results agree with the previous findings that a low NA is favorable for the welding process [10]. Another point to note is that, since the vortex beam has a higher effective beam area, the maximum breaking force is achieved with a higher input pulse energy, which induces higher thermal loads. The higher thermal loads can degrade the welding quality in return. In the loosely focused vortex beam condition, to achieve the maximum breaking force, an input pulse energy of 2.14 µJ is required. This high thermal load might counterbalance the potential improvement and explain the insignificant enhancement of the breaking force.

### 3.5. Welding of Different Material Pairs

Based on the investigations carried out in the previous section, to achieve maximum breaking force, we chose to use the loosely focused (4× MO) vortex beam for the welding in this section. The other processing parameters remain the same as in Section 3.4.

**Figure 8:**
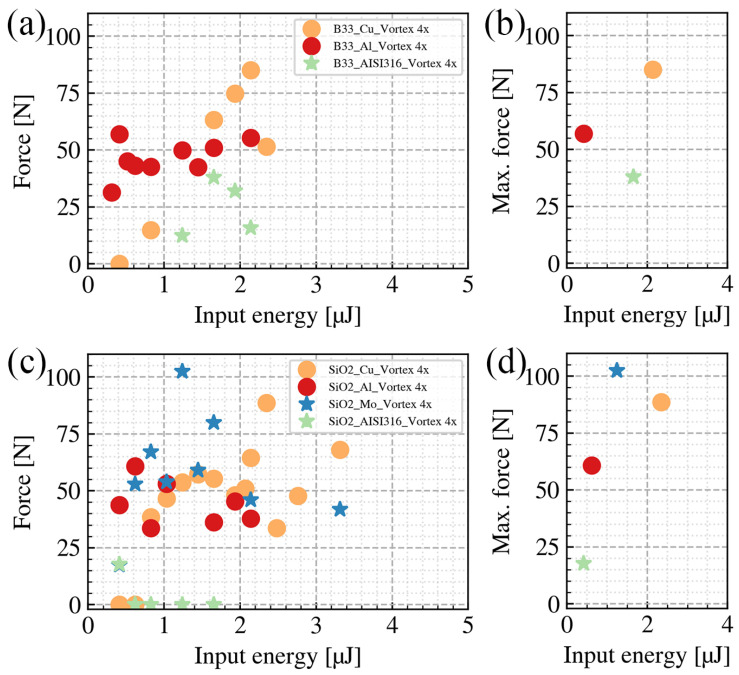
(**a**) Relationship between the breaking force and input pulse energy under different B33-to-metal welding conditions. (**b**) The maximum breaking force for each B33-to-metal condition. (**c**) Relationship between the breaking force and input pulse energy under different ${\mathrm{SiO}}_{2}$-to-metal welding conditions. (**d**) The maximum breaking force for each ${\mathrm{SiO}}_{2}$-to-metal condition. The processing area *A* is 4.5 mm × 2.0 mm.

We first investigated the weldability of B33 with three different metals (Copper, Aluminium, and AISI316 steel). In Figure 8a, the breaking force to input pulse energy relations under different B33-to-metals welding conditions are illustrated. The maximum breaking force for each condition is summarized in Figure 8b. Among all three metals, the B33-to-Cu pair achieved the highest breaking force (85.12 N) and the B33-to-AISI316 steel pair achieved the lowest breaking force (37.9 N). The maximum breaking force of B33-to-Al is 56.9 N. Compared to Cu and Al, AISI316 steel is more difficult to weld with B33 glass. It exhibits not only the lowest breaking force but also the smallest processing windows in terms of the input pulse energy, achieving welding only at pulse energies below (∼1 µJ).

Figure 8c,d present the ${\mathrm{SiO}}_{2}$-to-metal welding results. Similar trends are followed compared to B33-to-metal welding. The maximum breaking force of ${\mathrm{SiO}}_{2}$-to-Cu welding is 88.62 N, and for ${\mathrm{SiO}}_{2}$-to-Al welding it is 60.8 N. For ${\mathrm{SiO}}_{2}$, we conducted additional welding experiments using molybdenum. The maximum breaking force of ${\mathrm{SiO}}_{2}$-to-Mo welding is 102.4 N, which is the highest among all tested glass-to-metal welding cases. Similar to the B33 welding condition, AISI316 steel has the worst weldability with ${\mathrm{SiO}}_{2}$ as well. The maximum breaking force of ${\mathrm{SiO}}_{2}$-to-AISI316 is 17.7 N, and the processing windows are at pulse energies lower than 1 µJ. Successful welding of ${\mathrm{SiO}}_{2}$ to stainless steel was achieved in [17] at similar pulse energies. With a welding area that is about 75% compared to ours, they reach breaking forces of up to 240 N. They attribute the high stability of their welds to the high pressures used in their clamping system.

### 3.6. FEM Analysis of Thermal Stress

#### 3.6.1. Method

To understand the origin of the various welding qualities in different material pairs, a thermal-mechanical model based on the finite-element method was built to analyze the welding process. In this simulation model, we coupled a laser heating thermal diffusion model (details given in the Appendix B) with a structural mechanical model to simulate the von Mises stress (thermal stress) induced by the laser heating in different material pairs.

**Figure A1:**
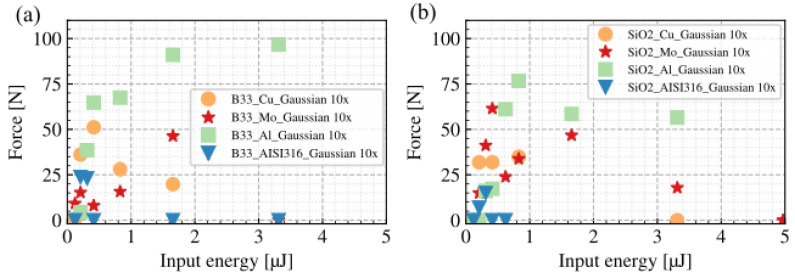
(**a**) Relationship between the breaking force and input pulse energy under different B33-to-metal welding conditions. (**b**) Relationship between the breaking force and input pulse energy under different ${\mathrm{SiO}}_{2}$-to-metal welding conditions. The processing area *A* is 4.5 mm × 2.0 mm.

As for our experiments in Section 3.5, the repetition rate of the laser is 330 kHz with four pulses per burst. Even though different metals have different reflectances, to focus on the thermal-mechanical properties of the welds, in the simulation, the absorbed pulse energy (nonlinear absorption $E_{nl}$ in the glass + linear absorption $E_{l}$ in the metal) was set at constant (64 nJ). Due to limited computational power, we only simulated the first 145 ns of the welding process. Within this period, the samples experienced only the first burst. For straightforward modeling, the beam shape was set to Gaussian, and the radius of the beam waist at the focus was 1.9 µm, corresponding to the tightly focused Gaussian beam (10× MO). The corresponding experimental data is given in the appendix, see Figure A1.

#### 3.6.2. Results

**Figure 9:**
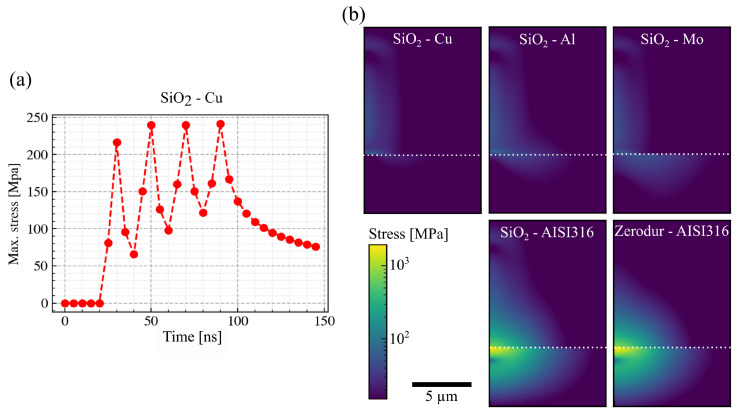
(**a**) Temporal evolution of the maximum thermal stress induced during ${\mathrm{SiO}}_{2}$-to-Cu welding. (**b**) Longitudinal cross-sections of the thermal stress distributions at the end of the simulated time period (t=145ns) for various material pairs. The dotted white line indicates the position of the interface.

As an example, the time-resolved thermal stress and the temporal evolution of the maximum thermal stress induced during the ${\mathrm{SiO}}_{2}$-to-Cu welding are presented in Figure 9a. The maximum thermal stress (241 MPa) was reached at the end of the fourth (last) pulse in the burst. At the end of the simulated time period, the thermal stress decreased to 76 MPa. The stress distribution at this moment (t=145ns) for different material pairs is presented in Figure 9b. The maximum thermal stresses are similar for ${\mathrm{SiO}}_{2}$-to-Cu/Al/Mo weldings (76 MPa, 78 MPa, 78 MPa). However, compared to Cu and Mo, ${\mathrm{SiO}}_{2}$-to-Al welding exhibits a larger thermal stress-affected area within the ${\mathrm{SiO}}_{2}$ (width of the stress distribution above the interface ∼5.6 µm vs. 2.6 µm). The large thermal stress-affected area may lead to the degradation of the welds.

The maximum thermal stress induced by ${\mathrm{SiO}}_{2}$-to-AISI316 is one magnitude higher (1801 MPa) than that induced in ${\mathrm{SiO}}_{2}$-to-Cu/Al/Mo pairs. According to our simulation model, such high thermal stress is due to the low thermal conductivity of the steel. Compared to Cu/Al/Mo, the absorbed laser energies are transferred away from the interface at a slower rate. Therefore, a higher thermal load is reached at the ${\mathrm{SiO}}_{2}$-to-AISI316 interface. The high thermal stress at the interface could help explain why AISI316 steel is difficult to weld with ${\mathrm{SiO}}_{2}$ glass. Titanium (Ti) also has a low thermal conductivity (see Table A1 for reference) and according to our simulation results, it also exhibits high thermal stress (same magnitude as AISI316). In our welding experiments, we did not achieve any welding between Titanium and either B33 or ${\mathrm{SiO}}_{2}$ glass, which seems to indicate that thermal stress caused by heat accumulation due to low thermal diffusion in those metals leads to a lower strength of the welding seams. Motivated by the high industrial relevance of stainless steel and titanium, we conducted further experiments with ZERODUR^®^ (SCHOTT AG., Minden, Germany), which are presented in the next section. The variation of thermal conductivity in the glasses is small compared to those in the metals and can be neglected. In further experiments it would also be interesting to examine the welding of glasses to a metal with similar coefficient of thermal expansion (CTE).

### 3.7. Welding of Glass to Metals with Low Thermal Conductivity

**Figure A2:**
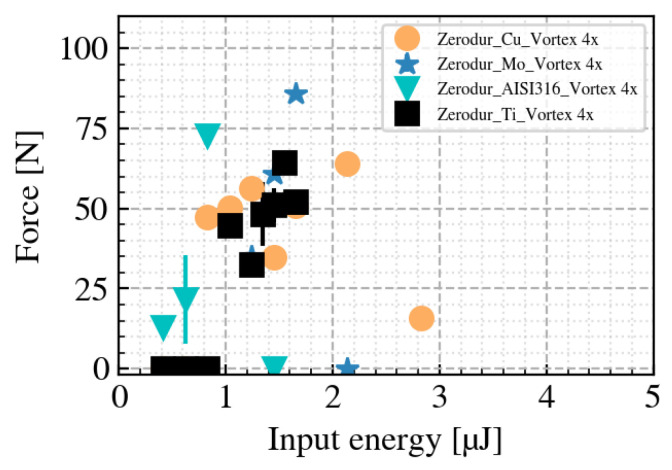
Relationship between the breaking force and input pulse energy under different Zerodur-to-metal welding conditions. The processing area *A* is 4.5 mm × 2.0 mm.

ZERODUR^®^ is a glass-ceramic with an extremely low coefficient of thermal expansion (CTE) of 0 ± 0.020 ppm/K in the temperature range 0 °C to 50 °C (also see Table A1 for reference). Using the same repetition rate, beam shape, and welding area (4.5 mm × 2 mm), the maximum breaking force reached for ZERODUR^®^-to-AISI6316 welding was 72.7 N, achieving an improvement of over 400% compared to the best ${\mathrm{SiO}}_{2}$-to-AISI316 result. The maximum breaking force for ZERODUR^®^-to-Titanium welding was 64.4 N, also a remarkably high value compared to our other welding strengths. The experimental results for ZERODUR^®^ are shown in the appendix, see Figure A2.

Perhaps surprisingly, the calculated thermal stresses for ZERODUR^®^ are similar to those of ${\mathrm{SiO}}_{2}$-to-AISI316, as you can see in the bottom row of Figure 9b. However, the height of the volume affected by the thermal load in ZERODUR^®^ is significantly smaller than that in ${\mathrm{SiO}}_{2}$, extending approximately 6 µm and 10 µm upwards from the interface, respectively. This can be readily explained in terms of the low CTE of ZERODUR^®^, which leads to a larger CTE mismatch at the interface, but also to a much smaller internal deformation due to the thermal load. This suggests that the critical damage mechanism of the welding seams is not the failure of the glass–metal connection but internal failure of the dielectric. This aligns with our previous findings, where we usually observed fragments of the glass on the metal surface when there was a measurable welding strength.

## 4. Conclusions

In this paper, we first examined the influence of pulse energy, longitudinal focus position, and line spacing on welding quality. Based on these results, we further investigated glass-to-metal welding with different beam shapes and material combinations. Compared to Gaussian beams, a vortex beam can enhance the breaking force of the welds. Using a loosely focused (4× MO) vortex beam, among different metal and glass pairs, the maximum breaking force was achieved in the ${\mathrm{SiO}}_{2}$-to-Mo welding. When the line spacing was set at 100 µm, the maximum breaking strength was calculated to be 11.4 MPa. We found that commonly used stainless steel and titanium struggle to weld with conventional glasses. Through our FEM analysis, we determined that the poor weldability is due to increased laser-induced thermal stress within the glass, caused by heat accumulation from the low thermal conductivity of those metals. Ultimately, we discovered that by using ZERODUR^®^ as a dielectric component, robust welds with stainless steel and titanium can be achieved, with more than a four-fold increase in strength compared to ${\mathrm{SiO}}_{2}$. The somewhat surprisingly high simulated peak thermal stresses for those very strong welds with ZERODUR^®^ may indicate internal failure of the dielectric as the critical failure mechanism for the welding seams. As this hypothesis cannot be conclusively confirmed based on our data, it would be interesting to examine the failure mechanisms further, e.g., by fractographic analysis of the separated samples or cross-sections of the welded samples, see [31].

## Data Availability

Data is contained within the article.

## Appendix A. Material Parameters

**Table A1:** Thermal and mechanical parameters of the materials investigated in the welding experiments.

	Thermal Conductivity(W/m·K)	Heat Capacity (J/kg·K)	Density (kg/$m^{3}$)	Melting Temperature (K)	Young’s Modulus (GPa)	Thermal Expansion (1/K)	Poisson’s Ratio
SiO_2_ *	1.40	730	2200	1956	70	0.5 × 10^−6^	0.17
B33 ^†^	1.20	830	2230	1093	64	3.25 × 10^−6^	0.20
Zerodur ^‡^	1.43	800	2530	N/A	90.3	0 ± 0.02 × 10^−6^	0.24
Aluminium	238	900	2700	934	70	23 × 10^−6^	0.33
Copper	400	385	8960	1358	110	17 × 10^−6^	0.35
Molybdenum	138	250	10200	2896	312	4.8 × 10^−6^	0.31
AISI316 Steel	15	500	8000	1370–1400	205	12.6 × 10^−6^	0.28
Titanium	7.5	710	4940	1941	105	7.06 × 10^−6^	0.33

* Fused silica UV-Grade. ^†^ SCHOTT BOROFLOAT^®^ 33 [32]. ^‡^ SCHOTT ZERODUR^®^ [33]. Sources for ${\mathrm{SiO}}_{2}$ and metals: COMSOL 6.0 material library, Goodfellow.

## Appendix B. Temperature Model

Even though a two-temperature model is required for a precise description of ultrashort pulse laser absorption [34], in this paper, our simulation uses a simple thermal diffusion model, assuming excitation of the material with a Gaussian pulse shape. As the pulse duration is much shorter than a time step in the simulation, the energy absorption by a laser pulse is assumed to be instantaneous. In our simulations, the input pulse energy $E_{0}$ is set to 200 nJ, and the absorptivity in glass $\alpha_{g}$ is estimated as 30%, which fits to the results presented in [21]. Given the approximate value of this absorptivty, we neglect the losses due to Fresnel reflection at the glass surface. For the absorptivity of the metal $\alpha_{m}$, we calculated the value of copper in terms of its reflectance $R_{c}$ at our laser wavelength, so that $\alpha_{m}=1-R_{c}=1-0.97151$. Thus, as input for our simulations, we obtain the absorbed energy of 60 nJ and 4 nJ in the glass and metal, respectively. In reality, even for a constant pulse energy, the nonlinear absorption in the glass and the linear absorption in the metal will differ for each material pair. However, since our focus is on highlighting the influence of mechanical properties, we deliberately keep these values constant across all material pairs. The heat transfer and the corresponding temperature distribution $T=T(\vec{r},t)$ are calculated with an explicit finite difference model. Among the three main mechanisms of heat transfer (heat conduction, heat convection, and thermal radiation), we focus on heat conduction as the primary mechanism of heat transfer in our model and neglect the other two. Also, we consider glass and metal to be in perfect thermal contact, although for a more detailed thermal analysis, we should also consider the effect of the gap on the heat transfer.

The governing equation in this model is then the heat transfer equation

(A1) $$\rho C_{p}\frac{\partial T}{\partial t}-\nabla \left(k\nabla T\right)=Q,$$

where ρ, $C_{p}$ and *k* are the temperature dependent material density, heat capacity and thermal conductivity of the glass and the metal respectively. The heat source *Q* of the heat transfer equation is the absorbed pulse laser energy per unit volume which can be written as

(A2) $$Q\left(r,z\right)=\left\{\begin{array}{c} \frac{\alpha_{g}E_{0}}{\tau \cdot l_{\mathrm{abs}}\cdot \omega \left(z\right)}\exp\left(\frac{-2r^{2}}{\omega {\left(z\right)}^{2}}\right);\;0\leq z\leq l_{\mathrm{abs}}\\ \frac{\alpha_{m}\left(1-\alpha_{g}\right)E_{0}}{\tau \cdot D_{p}\cdot \omega \left(z\right)}\exp\left(\frac{-2r^{2}}{\omega {\left(z\right)}^{2}}-\alpha_{m}\left|z\right|\right);\;z<\;0 \end{array},\right.$$

where $r=\sqrt{x^{2}+y^{2}}$, $D_{p}$ is the penetration depth of copper, and z=0 corresponds to the position of the glass-to-metal interface. By ignoring the spherical aberration induced at the air-to-glasss interface [30], as well as the nonlinear (Kerr) self-focusing and (plasma) defocusing [7], the beam radius of the laser beam propagating in the bulk of glass is given by

(A3) $$\omega \left(z\right)=\omega_{0}\sqrt{1+\left(\frac{M^{2}\lambda z}{\pi \omega_{0}^{2}n_{g}}\right)};\;\omega_{0}=\frac{M^{2}\lambda }{\pi N\;A_{\mathrm{obj}}}.$$

where $n_{g}$ is the refractive index of the glass.

$l_{\mathrm{abs}}$ is the length of the absorption region, according to Miyamoto et al. [21], and it can be estimated as

(A4) $$l_{\mathrm{abs}}=\frac{n_{g}}{N\;A_{\mathrm{obj}}}\sqrt{\frac{2E_{\mathrm{abs}}}{\pi \tau I_{\mathrm{th}}}-\omega_{0}^{2}},$$

where $I_{\mathrm{th}}$ is the damage threshold of the B33 glass.
